# Epstein-Barr virus antibodies in serum and DNA load in saliva are not associated with radiological or clinical disease activity in patients with early multiple sclerosis

**DOI:** 10.1371/journal.pone.0175279

**Published:** 2017-04-07

**Authors:** René M. Gieß, Catherina Pfuhl, Janina R. Behrens, Ludwig Rasche, Erik Freitag, Nima Khalighy, Carolin Otto, Jens Wuerfel, Alexander U. Brandt, Jörg Hofmann, Bettina Eberspächer, Judith Bellmann-Strobl, Friedemann Paul, Klemens Ruprecht

**Affiliations:** 1 Department of Neurology, Charité - Universitätsmedizin Berlin, Berlin, Germany; 2 NeuroCure Clinical Research Center, Charité - Universitätsmedizin Berlin, Berlin, Germany; 3 Clinical and Experimental Multiple Sclerosis Research Center, Charité - Universitätsmedizin Berlin, Berlin, Germany; 4 St. Josefs-Krankenhaus, Potsdam, Germany; 5 MIAC AG and Dep. for Biomedical Engineering, University of Basel, Basel, Switzerland; 6 Labor Berlin Charité-Vivantes GmbH, Berlin, Germany; 7 Institute of Medical Virology, Charité –Universitätsmedizin Berlin, Berlin, Germany; 8 Vivantes Klinikum Neukölln, Berlin, Germany; 9 Experimental and Clinical Research Center, Charité - Universitätsmedizin Berlin and Max Delbrück Center for Molecular Medicine, Berlin-Buch, Germany; United Arab Emirates University, UNITED ARAB EMIRATES

## Abstract

**Objective:**

To investigate the association of Epstein-Barr virus (EBV) nuclear antigen-1 (EBNA-1) and viral capsid antigen (VCA) immunoglobulin (Ig)G antibodies in serum as well as EBV DNA load in saliva with radiological and clinical disease activity in patients with clinically isolated syndrome (CIS) and early relapsing-remitting MS (RRMS).

**Methods:**

EBNA-1 and VCA immunoglobulin (Ig)G antibodies were determined in serum of 100 patients with CIS/early RRMS and 60 healthy controls. EBV DNA load was measured in saliva of 48 patients and 50 controls. Patients underwent clinical assessment with the Expanded Disability Status Scale (EDSS) and 3 Tesla magnetic resonance imaging at baseline and after a median of 20 months of follow-up (n = 63 for MRI, n = 71 for EDSS). The association of EBV parameters with occurrence of a second relapse, indicating conversion to clinically definite MS (CDMS), was evaluated over a median of 35 months of follow-up after the first clinical event (n = 89).

**Results:**

EBNA-1 IgG antibody frequency (*p* = 0.00005) and EBNA-1 and VCA IgG antibody levels (*p*<0.0001 for both) were higher in patients than in controls. EBV DNA load in saliva did not differ between groups. Neither EBV antibody levels nor DNA load in saliva were associated with baseline or follow-up number or volume of T2-weighted (T2w) or contrast enhancing lesions, number of Barkhof criteria or the EDSS, or with the number of new T2w lesions, T2w lesion volume change or EDSS change on follow-up. Likewise, levels of EBV IgG antibodies in serum and DNA load in saliva were not associated with conversion to CDMS.

**Conclusions:**

While these findings confirm the association of EBV infection with early MS, neither EBNA-1 nor VCA IgG antibodies in serum nor EBV DNA load in saliva were associated with radiological or clinical disease activity in patients with CIS/early RRMS. These data are compatible with the concept that EBV may be a trigger for MS acting very early during the development of the disease.

## Introduction

Infection with the Epstein-Barr virus (EBV) is a strong risk factor for multiple sclerosis (MS), a chronic inflammatory demyelinating disease of the central nervous system [1, 2]. EBV seroprevalence in patients with MS is practically universal, indicating that MS risk among EBV seronegatives is extremely low [1, 3–5]. Symptomatic primary EBV infection (infectious mononucleosis) increases the relative risk of MS about twofold [6]. Initially seronegative persons, which went on to develop MS, seroconverted to EBV positivity before the onset of MS [7]. Among healthy individuals infected with EBV, MS risk increases with increasing serum titers of antibodies to Epstein-Barr nuclear antigen-1 (EBNA-1) and the EBV nuclear antigen complex (EBNAc) [8–12]. Accordingly, patients with MS or with a clinically isolated syndrome (CIS, i.e. a first clinical event suggestive of MS) have elevated levels of antibodies against EBV, in particular against EBNA-1 [13–18].

Although the association of EBV and MS risk is thus very robust, the association of parameters of EBV infection, such as serum levels of immunoglobulin (Ig)G antibodies to EBV, with disease activity in patients with CIS or MS is less clear-cut. While previous reports described associations of EBV antibodies in serum with certain radiological and clinical markers of MS disease activity, results were not always consistent across those studies [15, 19–26]. Furthermore, a recent large prospective study did not identify an association of EBV IgG antibodies in serum with risk of CIS conversion to MS, or MS activity or progression [27].

EBV is transmitted via oral secretions, enters through the epithelium that lines the oropharynx (Waldeyer’s ring) and infects naïve B cells, which differentiate through a germinal center reaction into memory B cells, where EBV persists for the lifetime of the host [28]. Upon returning of EBV-infected memory B cells to the oropharynx these can differentiate into plasma cells that subsequently initiate viral replication and release virions that are shed into the saliva [29]. However, the role of EBV DNA load in saliva as a biomarker for disease activity in adults with early MS has not been examined so far.

In this study, we investigated whether EBNA-1 and viral capsid antigen (VCA) IgG antibody levels in serum, as well as EBV DNA load in saliva, are associated with clinical and magnetic resonance imaging (MRI) markers of disease activity, severity and progression in a cohort of 100 patients with CIS or early relapsing-remitting MS (RRMS).

## Patients and methods

The study was approved by the institutional review board of Charité - Universitätsmedizin Berlin (EA1/182/10). All participants provided written informed consent.

### Patients and healthy controls

The Berlin CIS cohort (NCT01371071) is an ongoing prospective observational study of patients with a first clinical event suggestive of inflammatory demyelination (i.e. a CIS [30]) or early RRMS that started recruitment in January 2011. Inclusion criteria are age >18 years, a first clinical event suggestive of central nervous system demyelination within 6 months before inclusion into the study or a diagnosis of RRMS according to the McDonald 2010 criteria [31] within 24 months before inclusion into the study. Exclusion criteria are inability or unwillingness to provide informed consent, a history of alcohol or drug abuse, any ocular diseases precluding performance of optical coherence tomography, and any conditions (e.g. allergies) or devices (e.g. cardiac pacemaker) precluding MRI examinations. At the baseline visit, all patients underwent a thorough clinical assessment, including Expanded Disability Status Scale (EDSS) score, and were studied by cerebral and spinal MRI. Additionally, serum, and whole blood samples were obtained by peripheral venipuncture and about 1 ml of saliva was collected in sterile cups. In 85 of 99 (86%) patients who underwent MRI blood and saliva was withdrawn on the day of the MRI examination. The median (range) delay between the blood/saliva withdrawal and MRI in the remaining 14 patients was 9 (1–49) days. Clinical and MRI assessments were repeated on follow-up visits every 12 months after onset of first clinical symptoms. Serum and saliva samples were also obtained from 60 healthy controls, matched by sex and age to the first 60 patients with CIS/RRMS enrolled in the study. All serum samples were processed at the NeuroCure Clinical Research Center, Charité –Universitätsmedizin Berlin, according to standard operating procedures. Saliva samples and aliquoted sera were stored at -20°C and -80°C, respectively.

### Magnetic resonance imaging

High-resolution three-dimensional isotropic whole brain datasets (1mm^3^) were acquired on a 3 Tesla whole-body MRI (Magnetom Trio with TIM, Siemens Healthcare AG, Erlangen, Germany), using a clinical routine 12-channel head coil. For anatomical T1-weighted imaging, a magnetization-prepared rapid acquisition and multiple gradient echo technique (MPRAGE, TE 3.03 ms, TR 1900 ms, TI 900 ms, flip angle 9°) was applied. For T2-weighted imaging (T2w), a single slab three-dimensional T2w turbo-spin-echo (TSE) sequence with high sampling efficiency (SPACE) was selected without (T2; TE 388 ms, TR 6000 ms, flip angle 120°) or with fluid inversion recovery pulce (FLAIR; TE 502 ms, TR 5000 ms, TI 2100 ms, flip angle 120°). To rule out brain stem and infratentorial artifacts, an axial double-echo proton density/T2w sequence was added (TE 14/87 ms, TR 3400 ms, flip angle 120°, voxel resolution 1 x 1 x 3 mm^3^, no gap). Contrast enhanced images were acquired by a volumetric interpolated brain examination sequence optimized for short acquisition time with asymmetric k-space sampling and interpolation (VIBE; 1 mm^3^, TE 2.2 ms, TR 4.85 ms, flip 9°) 8 min after body weight adapted 1 mmol Gadubutrol injection). The number of T2w lesions, contrast-enhancing lesions (CEL), and number of Barkhof criteria were scored. CEL as well as T1w and T2w lesion load was calculated using the OsiriX software toolbox (OsiriX foundation, Geneva, Switzerland) and in-house applications. All MRI data were analyzed by investigators blinded to the results of laboratory studies.

### Anti-EBV antibodies

Serum immunoglobulin (Ig)G antibodies to EBNA-1 and to the EBV viral capsid antigen (VCA) were measured by Liaison® (DiaSorin, Saluggia, Italy) automated chemiluminescent assays at Labor Berlin GmbH in serum samples collected at the baseline visit. According to the manufacturer’s recommendations, EBNA-1 IgG levels <5 U/ml were considered negative, levels between 5–20 U/ml were considered equivocal, and levels ≥20 U/ml were considered positive. VCA IgG levels <20 U/ml were considered negative and VCA IgG levels ≥20 U/ml were considered positive. Samples with values above the upper detection limit were re-measured at a dilution of 1:20, as suggested by the manufacturer. The upper detection limit following dilution was 12,000 U/ml for EBNA-1 IgG and 15,000 U/ml for VCA IgG. Persons with positive antibodies to either EBNA-1 or VCA or both were considered EBV-seropositive. EBNA-1- and VCA-IgG-negative persons were considered EBV-seronegative.

### Quantitation of EBV-DNA in whole blood and saliva samples

EBV DNA was quantitated in whole blood and saliva collected at the baseline visit by a real time PCR for the BNRF1 p143 gene using primers and probes described elsewhere [32]. DNA was extracted from 200 μl of whole blood or undiluted saliva using the QIAamp DNA Mini Kit (Qiagen, Germany) according to the manufacturer’s instructions. All samples were controlled for the presence of inhibiting factors by the use of an internal co-amplified DNA. The 95% detection limit of the assay is 1000 copies/ml. In case small amounts of EBV DNA below this level could be detected these were reported as <1000 EBV DNA copies/ml.

### Statistical analysis

Statistical significance of frequencies was assessed by Fisher’s exact test. Significance of different antibody levels and DNA load in saliva in patients and controls was assessed by Mann Whitney test. Significance of different antibody levels in the groups of untreated, glatiramer-acetate-treated, and interferon-beta-treated patients with MS were assessed by Kruskal-Wallis test. Associations between EBV parameters and clinical (EDSS) and MRI disease activity measures at baseline and a minimum of 12 months of follow-up were assessed by Spearman correlations. Associations of EBV parameters with conversion to clinically definite MS (CDMS) were assessed by Kaplan-Meier curves in all patients with only one clinical event before inclusion into the cohort. For Kaplan-Meier analyses, patients were grouped into tertiles of low, medium and high EBNA-1 or VCA IgG levels in serum as well as into those with and without EBV DNA in saliva. All statistical analyses were performed with GraphPad Prism Version 6.01. *P*-values <0.05 were considered statistically significant.

## Results

### Participants

The demographics, baseline EDSS values, immunomodulatory treatments and baseline MRI findings of the 100 patients with CIS (n = 76) or early RRMS (n = 24) analyzed in this study were typical of patients with early MS and are summarized in Table 1. While the gender distribution was not significantly different between the patient and the healthy control group (*p* = 0.13), healthy controls were on average about 3 years younger than CIS/RRMS patients (*p* = 0.01). Of the 24 patients with a diagnosis of RRMS, 11 had experienced two relapses prior to inclusion into the cohort, thus meeting criteria for clinically definite MS (CDMS), and 13 had a diagnosis of RRMS based on one clinical relapse and fulfilment of MRI criteria for RRMS [31]. Thus, 89 patients had experienced one single event suggestive of inflammatory demyelination at the time of entering the prospective cohort.

**Table 1:** Demographics, clinical and MRI findings and EBV parameters at baseline of patients and healthy controls analyzed in this study.

	CIS (n = 76)	Early RRMS (n = 24)	Controls (n = 60)
Females/Males (% Female)	53/23 (70)	15/9 (62.5)	33/27 (55)
Median (range) age, years	32 (19–56)	30 (18–53)	27 (20–54)
Median (range) EDSS	1.5 (0–3.5)	1.5 (0–4)	n.a.
Immunomodulatory treatment (number)	• None (58) • Glatiramer acetate (6) • Interferon-beta-1a i.m. (3) • Interferon-beta-1a s.c. (7) • Interferon-beta-1b s.c. (2)	• None (12) • Glatiramer acetate (8) • Interferon-beta-1a i.m. (2) • Interferon-beta-1a s.c. (1) • Immunoglobulins i.v. (1)	n.a.
Mean (SD) number T2w lesions	15.6 (25.5)	31.7 (38.8)	n.a.
Mean (SD) T2w lesion volume (cm^3^)	1.3 (2.3)	3 (3.7)	n.a.
Mean (SD) CEL number	0.03 (0.18)	1.5 (1.8)	n.a.
Mean (SD) CEL volume (cm^3^)	0.0012 (0.007)	0.14 (0.3)	n.a.
0 BC, number (%)	14 (19)	1 (4)	n.a.
1–2 BC, number (%)	30 (41)	5 (22)	n.a.
3–4 BC, number (%)	29 (40)	17 (74)	n.a.
Mean (SD) Anti-EBNA1 IgG (AU/ml)	1120 (1530)	750 (1230)	485.7 (972.9)
Mean (SD) Anti-VCA IgG (AU/ml)	498.2 (468.3)	449.1 (492.7)	283.4 (481.2)
Mean (SD) EBV DNA (copies/ml)	32713 (98294) n = 37	164107 (448831) n = 11	28370 (107671) n = 47

AU = arbitrary units, BC = Barkhof criteria, CEL = contrast enhancing lesions, CIS = clinically isolated syndrome, EBNA-1 = Epstein-Bar nuclear antigen-1, EDSS = Expanded Disability Status Scale, RRMS = relapsing-remitting multiple sclerosis, SD = standard deviation, VCA = viral capsid antigen

### EBV seroprevalence and EBNA-1 and VCA IgG antibody levels in patients and controls

**Fig 1:**
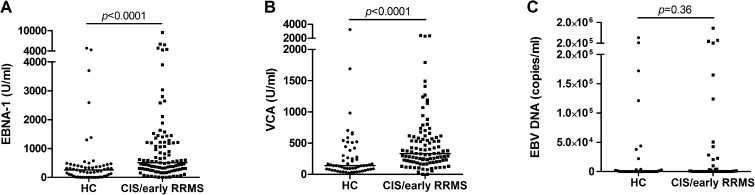
EBV antibodies in serum and DNA load in saliva in healthy controls and patients with early MS. Levels of Immunoglobulin (Ig)G antibodies to Epstein-Barr nuclear antigen-1 (EBNA-1, A) and viral capsid antigen (VCA, B) in serum of healthy controls (HC, n = 57) and patients with clinically isolated syndrome (CIS) and early relapsing-remitting MS (RRMS, n = 100). (C) EBV DNA copy number in saliva of healthy controls (n = 47) and patients with CIS/early RRMS (n = 48). The horizontal lines indicate the median (median EBV DNA load was 0 in HC and CIS/early RRMS).

IgG antibodies to EBNA-1 above the cut-off level of 20 U/ml were detectable in 96/100 (96%) of patients with CIS/RRMS and 44/60 (73%) of healthy controls (*p* = 0.00005). VCA IgG antibodies above the cut-off level of 20 U/ml were detected in 98/100 (98%) patients with CIS/RRMS and 57/60 (95%) of healthy controls (*p* = 0.36). All EBNA-1 IgG seronegative patients had antibodies to VCA and all VCA IgG seronegative patients had antibodies to EBNA-1. However, the three VCA IgG seronegative healthy controls neither had antibodies to EBNA-1. Thus, all patients with MS (100%) but only 57/60 (95%) of the healthy controls had serologic evidence of prior EBV infection (*p* = 0.051). EBNA-1 and VCA IgG antibody levels were significantly higher (*p*<0.0001 for both) in patients with CIS/RRMS (n = 100) than in EBV-seropositive healthy controls (n = 57; Fig 1). There were no significant differences of EBNA-1 (*p* = 0.07) or VCA (*p* = 0.42) antibody levels in patients with CIS or RRMS. EBNA-1 (*p* = 0.39) and VCA (*p* = 0.67) antibody levels did not differ between the groups of untreated (n = 70), glatiramer acetate-treated (n = 14), and interferon-beta-treated (n = 15) patients with CIS/early RRMS.

### EBNA-1 and VCA antibodies and radiological and clinical markers of disease activity

At baseline, neither EBNA-1 nor VCA antibody levels showed any significant associations with the number or volume of T2 lesions, number or volume of CEL, or the number of Barkhof criteria (Table 2). Neither EBNA-1 nor VCA IgG antibodies correlated with EDSS at baseline or age at first symptoms.

**Table 2:** Association of EBV parameters with radiological and clinical disease activity at baseline.

	Number of T2w lesions (baseline)	Total volume of T2w lesions (baseline)	Number of contrast-enhancing lesions (baseline)	Total volume of contrast-enhancing lesions (baseline)	Number of Barkhof criteria (baseline)	EDSS (baseline)	Age at onset of first symptoms
Anti-EBNA1 IgG	• n = 99 • *r* = 0.003 • *p* = 0.978	• n = 99 • *r* = 0.004 • *p* = 0.972	• n = 82 • *r* = -0.066 • *p* = 0.558	• n = 82 • *r* = -0.041 • *p* = 0.713	• n = 96 • *r* = 0.036 • *p* = 0.728	• n = 100 • *r* = -0.196 • *p* = 0.051	• n = 100 • *r* = 0.148 • *p* = 0.141
Anti-VCA IgG	• n = 99 • *r* = 0.074 • *p* = 0.465	• n = 99 • *r* = 0.045 • *p* = 0.660	• n = 82 • *r* = -0.083 • *p* = 0.456	• n = 82 • *r* = -0.064 • *p* = 0.568	• n = 96 • *r* = 0.071 • *p* = 0.494	• n = 100 • *r* = 0.007 • *p* = 0.945	• n = 100 • *r* = 0.145 • *p* = 0.151
EBV DNA (saliva)	• n = 48 • *r* = 0.082 • *p* = 0.577	• n = 48 • *r* = 0.052 • *p* = 0.724	• n = 41 • *r* = 0.036 • *p* = 0.823	• n = 41 • *r* = 0.102 • *p* = 0.527	• n = 48 • *r* = 0.131 • *p* = 0.374	• n = 48 • *r* = 0.154 • *p* = 0.294	• n = 48 • *r* = 0.028 • *p* = 0.848

Association of baseline EBNA-1 and VCA IgG levels in serum and EBV DNA load in saliva with radiological and clinical disease activity markers at baseline.

EBV = Epstein-Barr virus, EBNA-1 = Epstein-Barr nuclear antigen-1, EDSS = Expanded Disability Status Scale, IgG = immunoglobulin G, n = number of pairs, *r* = Spearman’s r, T2w = T2-weighted, VCA = viral capsid antigen

Follow-up 3T MRI data, obtained at least 12 months after the baseline visit (median 20, range 12 to 29 months), was available from a total of 63/100 patients. 41 of these 63 patients showed new T2w lesions on follow-up MRI (median 5, range 1 to 47 new lesions). There were no significant associations between baseline EBNA-1 or VCA IgG antibody levels and the number or volume of T2w or CEL, the number of new T2w lesions, the volume change of T2w lesions and the number of Barkhof criteria on follow-up MRIs.

Follow-up EDSS data, obtained at least 12 months after the baseline visit (median 20, range 12 to 29 months), was available from a total of 71/100 patients. Neither EBNA-1 nor VCA IgG antibody levels were associated with the EDSS or changes in the EDSS (EDSS on follow-up minus EDSS at baseline) on follow-up (Table 3).

**Table 3:** Association of EBV parameters with radiological and clinical disease activity on follow-up.

	Number of T2w lesions (follow-up)	Total volume of T2w lesions (follow-up)	Number of contrast-enhancing lesions (follow-up)	Total volume of contrast-enhancing lesions (follow-up)	Number of new T2w lesions (follow-up)	Volume change in T2w lesions (follow-up)	Number of Barkhof criteria (follow-up)	EDSS (follow-up)	EDSS change (follow-up)
Anti-EBNA1 IgG	• n = 63 • *r* = -0.097 • *p* = 0.448	• n = 63 • *r* = -0.134 • *p* = 0.295	• n = 58 • *r* = -0.109 • *p* = 0.414	• n = 58 • *r* = -0.124 • *p* = 0.356	• n = 63 • *r* = -0.148 • *p* = 0.246	• n = 63 • *r* = -0.121 • *p* = 0.345	• n = 63 • *r* = -0.010 • *p* = 0.939	• n = 71 • *r* = -0.196 • *p* = 0.101	• n = 71 • *r* = -0.052 • *p* = 0.669
Anti-VCA IgG	• n = 63 • *r* = 0. 073 • *p* = 0.568	• n = 63 • *r* = 0.022 • *p* = 0.863	• n = 58 • *r* = -0.243 • *p* = 0.066	• n = 58 • *r* = -0.248 • *p* = 0.060	• n = 63 • *r* = -0.025 • *p* = 0.849	• n = 63 • *r* = -0.064 • *p* = 0.617	• n = 63 • *r* = 0.007 • *p* = 0.954	• n = 71 • *r* = 0.122 • *p* = 0.312	• n = 71 • *r* = 0.090 • *p* = 0.455
EBV DNA (saliva)	• n = 28 • *r* = -0.012 • *p* = 0.951	• n = 28 • *r* = -0.067 • *p* = 0.735	• n = 24 • *r* = 0.038 • *p* = 0.860	• n = 24 • *r* = 0.038 • *p* = 0.860	• n = 28 • *r* = -0.102 • *p* = 0.604	• n = 28 • *r* = 0.016 • *p* = 0.935	• n = 28 • *r* = 0.010 • *p* = 0.959	• n = 32 • *r* = 0.300 • *p* = 0.096	• n = 32 • *r* = -0.021 • *p* = 0.907

Association of baseline EBNA-1 and VCA IgG levels in serum and EBV DNA load in saliva with radiological and clinical disease activity markers at follow-up examinations performed a median of 20 (range 12 to 29) months after baseline examination.

EBV = Epstein-Barr virus, EBNA-1 = Epstein-Barr nuclear antigen-1, EDSS = Expanded Disability Status Scale, IgG = immunoglobulin G, n = number of pairs, *r* = Spearman’s r, T2w = T2-weighted, VCA = viral capsid antigen

### EBV DNA load in whole blood

EBV DNA load was measured in whole blood samples of 82 patients with CIS/RRMS. One sample could not be evaluated for technical reasons. In only 2 of the remaining 81 patients (2.5%) small amounts of EBV DNA (<1000 copies/ml) could be detected. The low number of EBV DNA positive plasma samples precluded correlations with clinical or MRI markers of disease activity.

### EBV DNA in saliva of patients and controls

EBV DNA load in saliva was determined in 48 patients with CIS/RRMS and 50 healthy controls. The age (*p* = 0.63) and sex (*p* = 1) distribution did not differ between both groups. There were 3 EBV seronegative persons (see above) among the 50 healthy controls, who expectedly had no EBV DNA in their saliva. These 3 EBV seronegative healthy controls were excluded from further evaluations. EBV DNA above the cut-off of 1000 copies/ml was detectable with a similar frequency in saliva of patients with CIS/RRMS (18/48, 37.5%) and of healthy controls (14/47, 30%; *p* = 0.52). Likewise, although quite substantial numbers of EBV DNA copies (maximum 1,506,000 copies/ml) could be detected in some individuals, the absolute amounts of EBV DNA in patients with CIS/RRMS and healthy controls did not differ (*p* = 0.36; Fig 1). Neither in patients with MS nor in healthy controls did salivary EBV DNA load show any associations with levels of antibodies to EBNA-1 (patients: *r* = 0.069, *p* = 0.64; controls: *r* = 0.232, *p* = 0.116) or VCA (patients: *r* = 0.076, *p* = 0.61; controls: *r* = 0.11, *p* = 0.475).

### EBV DNA load in saliva and markers of radiological and clinical disease activity

EBV DNA load in saliva was not associated with the number or volume of T2w or CEL, the number of Barkhof criteria, the EDSS or the age at baseline examination (Table 2). Likewise, EBV DNA load in saliva was not associated with the number or volume of T2w or CEL, the number of new T2w lesions, the volume change of T2w lesions on follow-up MRIs and the EDSS or the EDSS change on follow-up (Table 3).

### EBV parameters and conversion to clinically definite MS

**Fig 2:**
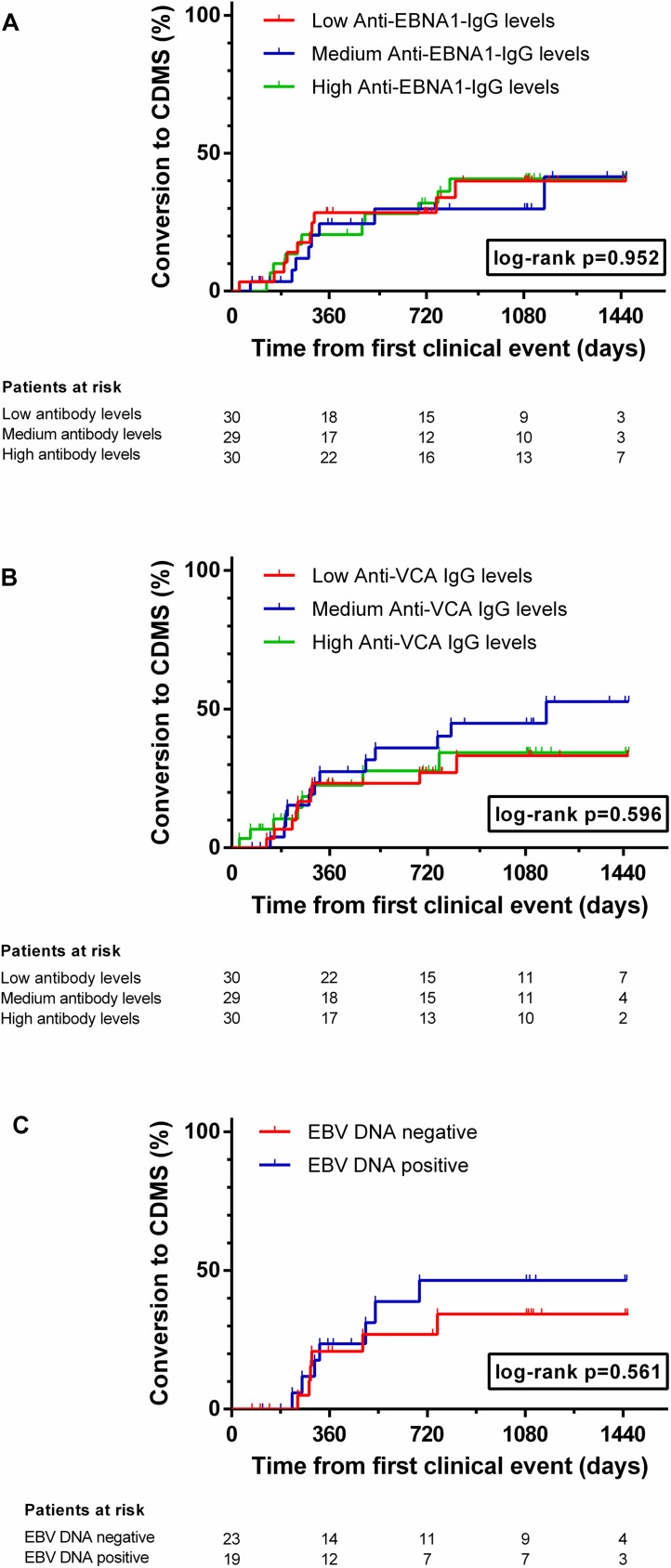
EBV antibodies in serum and DNA load in saliva and risk of conversion to CDMS. Risk of conversion to clinically definite multiple sclerosis (CDMS; Kaplan-Meier curves) by tertiles of immunoglobulin (Ig)G antibodies to Epstein-Barr nuclear antigen 1 (EBNA-1, A) or viral capsid antigen (VCA, B) and presence or absence of Epstein-Barr virus (EBV) DNA in saliva (C).

We next analyzed whether parameters of EBV infection might predict the occurrence of a second relapse, and thus conversion to CDMS, in all 89 patients who had only one clinical event before inclusion into the study. The median (range) follow-up time from onset of the first clinical event was 35 (2–51) months. During this period, 28 of 89 patients (31.5%) experienced a second relapse. A cut-off for the analysis was set at 4 years (1460 days) after the first clinical event. Patients were split into tertiles of low, medium or high serum levels of EBNA1 IgG or VCA IgG or were dichotomized into those with and those without EBV DNA in saliva. Neither EBNA1 IgG (*p*_log-rank_ = 0.952) nor VCA IgG (*p*_log-rank_ = 0.596) levels in serum nor presence of EBV DNA in saliva (*p*_log-rank_ = 0.561) were associated with conversion to CDMS (Fig 2).

## Discussion

In this prospective study of 100 patients in the earliest clinical stages of MS, neither EBNA-1 nor VCA IgG antibody levels in serum nor EBV DNA load in saliva were associated with established MRI and clinical markers of disease activity at baseline and over a median of 20 months of follow-up. Likewise, EBNA-1 and VCA IgG antibodies as well as EBV DNA load in saliva were not associated with conversion to CDMS over a median follow-up of 35 months after the first clinical event.

These findings are consistent with results from a large prospective study of 448 patients enrolled in the BENEFIT (Betaferon/Betaseron in Newly Emerging Multiple Sclerosis for Initial Treatment) trial, in which no association of EBNA-1 or VCA IgG levels in serum with conversion to CDMS, MRI activity markers of MS or clinical progression could be observed over a 5-year follow-up [27]. Similarly, in a large multicentre study of 1047 patients with a CIS, baseline levels of EBNA-1 IgG were not associated with T2w lesion load and were not predictive of conversion to CDMS over two years [33]. Furthermore, a prospective cohort study of 198 patients with MS found no associations between anti EBV early antigen (restricted) IgG, an EBV reactivation parameter, with subsequent MS relapse hazard or progression in disability [34]. Finally, another previous study did not observe correlations between EBNA-1 IgG in serum and EDSS or age at onset in patients with RRMS (n = 50) and primary progressive MS (PPMS, n = 25) [35].

In contrast, other studies have observed positive associations [15, 19–25, 36]. Among the studies that, similar to ours, focused on patients with CIS/early MS [15, 22, 23], a prospective study of 147 patients with a CIS found associations of EBNA-1 IgG in serum with the number of T2w lesions and Barkhof criteria at baseline, and with the number of T2w lesions, number of new T2w lesions and the EDSS after 1 and 5 years of follow-up, while EBNA-1 antibodies were not associated with conversion to CDMS in a multivariate model [15]. Likewise, and similar to our findings, in 211 interferon-beta treated patients with CIS enrolled in the SET study, EBNA-1 and VCA antibodies were not associated with conversion to CDMS over two years. However, unlike our findings, VCA IgG antibodies were associated with EDSS progression and the cumulative number of CEL and T2w lesions [23]. Conversely, another study of 50 patients with CIS, 25 with RRMS and 25 with PPMS observed associations of EBNA-1 IgG, but not of VCA IgG, with the number of CEL, change in T2w lesion volume and EDSS. Furthermore, CIS patients converting to CDMS within 5 years had higher levels of antibodies to EBNA-1 [22]. However, a recent cross-sectional study involving 539 patients with MS and 66 patients with CIS observed no associations of EBNA-1 IgG with any of the investigated MRI parameters, but again found associations of VCA IgG with T2w lesion volume and the number and volume of CEL in patients with MS, but not with CIS [26]. Nevertheless, a study that followed 87 patients with RRMS over two years showed no significant association of VCA IgG with MRI measures, but an association between EBNA-1 IgG in serum and the sum of CEL and new or enlarging T2w lesions [24].

Reasons for the diverging findings across studies may include different clinical and paraclinical characteristics of the analyzed cohorts, in particular rates and types of immunomodulatory treatment, or differences in the rates of patients with established parameters for MS disease activity, such as the presence of supernumerary oligoclonal bands in cerebrospinal fluid [37]. Furthermore, lack of correction of *p*-values for multiple testing in some of the studies may have resulted in a bias for positive associations. Finally, it has previously been pointed out that because of the elevated EBNA-1 IgG levels in patients with CIS/RRMS as compared to healthy controls (see Fig 1), inclusion in cohorts of patients with CIS of even few individuals who do not have MS and who thus will not have MRI or clinical disease activity on follow-up, will likely cause a spurious association between EBNA-1 levels and MS disease activity parameters [27]. Altogether, our present study confirms and reproduces previous findings, including those of 2 large previous prospective studies [27, 33], in an independent cohort, further corroborating the notion that IgG antibodies to EBV in serum are not associated with disease activity in early MS. This and the overall heterogeneous results of previous studies that described positive associations likewise suggest that EBNA-1 and VCA IgG levels in serum do not have the potential to serve as clinically meaningful disease activity biomarkers in patients with CIS/early RRMS.

To the best of our knowledge, this is the first study to systematically compare EBV DNA levels in saliva in adult patients with CIS/early RRMS and healthy controls. The similar frequencies and levels of EBV DNA in saliva of patients and controls do not support the hypothesis that a dysregulated EBV infection in patients with early MS may result in increased EBV DNA levels in saliva. A pediatric study detected EBV DNA more frequently in mouth swabs of children with MS (10/17; 59%) than of healthy pediatric controls (7/35; 20%) [38]. Reasons for the different findings may include different case numbers, patient age, and sampling techniques (saliva vs. mouth swabs). While a former study suggested an association of both EBV and human herpesvirus-6B DNA in saliva with clinical disease activity [39], the lack of an association of EBV DNA in saliva with disease activity in the present study suggests that, similar to EBV antibodies in serum, EBV DNA in saliva does not appear to be a promising disease activity biomarker for MS.

In keeping with previous findings [20, 22, 40, 41], EBV DNA was only very rarely detectable in whole blood of patients with CIS/early RRMS, again arguing against an impaired immunological control of EBV in patients with early MS.

The absence of significant associations between EBV antibodies and DNA in saliva with MS disease activity suggests that those parameters are not directly involved in the processes that determine radiological and clinical disease activity once the disease is established. Nevertheless, consistent with previous work [1, 3–5, 7], EBV seroprevalence in our cohort of patients in the earliest clinical phase of MS was 100%. Furthermore, and again consistent with previous work [13–18], levels of EBNA-1 and VCA IgG antibodies in serum were higher in patients with CIS/early RRMS than in healthy controls. These findings are compatible with the concept that EBV infection may be involved in the aetiology of MS at a very early point in time during development of the disease [42, 43]. While the precise mechanism underlying the association of EBV and MS currently remains unknown, future concepts and studies aiming to clarify this mechanism should therefore take into account that EBV may exert its role during the earliest phase of MS.

Among the strengths of this study are its prospective design, the comprehensive acquisition of clinical and 3T MRI data and the highly standardized biospecimen collection and storage. A limitation of our study is that MRI and EDSS follow-up data of at least 12 months were not available from all patients. Still, as the lack of an association of EBV parameters with follow-up MRI and EDSS data was very consistent, we consider it unlikely that our results would change substantially in a larger sample. This conclusion is likewise suggested by the study of Munger et al., which did not observe associations of EBV IgG with MRI activity measures on follow-up in a large number (n = 448) of patients. While we did not detect an association of EBV DNA load in saliva and whole blood with MS disease activity, we cannot exclude that EBV reactivation in secondary lymphoid organs or within the brain could be associated with disease activity in MS. Nevertheless, patients with MS do not show increased frequencies or levels of EBV DNA in their cerebrospinal fluid [40, 41], rather arguing against the possibility that EBV reactivation in the CNS may predict MS disease activity. As we measured EBV IgG in serum and DNA in saliva only at a single point in time, our study does not permit to draw conclusions on the temporal dynamics of EBV parameters (i.e. increase or decreases over time) and disease activity in MS. Finally, we cannot exclude that other parameters of EBV infection not addressed in the present work, such as cellular immune responses to EBV [44], might be associated with MS disease activity.

Altogether, this prospective study indicates that in patients with early MS levels of EBNA-1 and VCA IgG in serum and of EBV DNA in saliva are not associated with MRI and clinical markers of disease activity or with conversion to CDMS. Thus, these parameters of EBV infection do not appear to be promising disease activity biomarkers in patients with early MS. The universal EBV seroprevalence and an elevated humoral immune response to EBV in patients with CIS/early RRMS are compatible with the concept that EBV is involved in MS at a very early point in time during development of the disease.
